# Consumer Understanding of the Australian Dietary Guidelines: Recommendations for Legumes and Whole Grains

**DOI:** 10.3390/nu14091753

**Published:** 2022-04-22

**Authors:** Gynette Reyneke, Jaimee Hughes, Sara Grafenauer

**Affiliations:** 1 School of Medicine, University of Wollongong, Northfields Avenue, Wollongong, NSW 2522, Australia; glr603@uowmail.edu.au; 2 Grains & Legumes Nutrition Council, 1 Rivett Road, North Ryde, NSW 2113, Australia; 3 Faculty of Medicine and Health, School of Health Sciences, University of New South Wales, Randwick, NSW 2031, Australia; s.grafenauer@unsw.edu.au

**Keywords:** legumes, whole grains, consumer preferences, dietary guidelines, nutrition communication

## Abstract

Dietary guidelines provide evidence-based guidance for healthy individuals to improve dietary patterns, although they are most often based on individual foods or food groups. Legumes are a class of food included in current Australian Dietary Guidelines (ADG), mentioned in two of the five food groups, as a vegetable and as an alternative to meat. Whole grain consumption is encouraged in ADG via the statement focused on cereal grains due to their health-promoting properties. Despite their prominence in guidelines, average legume and whole grain consumption in Australia remains lower than recommendations outlined in the ADG. This exploratory study aimed to understand consumer perspectives of wording utilised in dietary guidelines specifically focused on legumes and whole grains. Based on the analysis, there was a significant preference for the statement “each day, consume at least one serve of legumes either as a serve of vegetables or as an alternative to meat” (*p* < 0.05), which provides a specific frequency and quantification for legume consumption. For whole grain, the significantly preferred statement was “choose whole grain products over refined grains/white flour products whenever you can” indicating a less prescriptive option. Effective messaging in guidelines could consider greater specificity regarding frequency, quantity and quality of foods recommended. This exploratory study suggests an improvement in the adoption and consumption of legumes and whole grains in the Australian diet may be better facilitated through consumer-tested messaging.

## 1. Introduction

Food based dietary guidelines provide evidence-based practical and actionable recommendations that aim to influence the dietary behaviours of the nation through consumer education and targeted health policies and programs [1]. The placement and classification of specific foods within the dietary guidelines are based upon the traditional dietary pattern of that country [1,2]. Consistent with a majority of other countries, the Australian Dietary Guidelines (ADG) promote proportional consumption of a variety of foods across five food groups: (i) vegetables; (ii) fruit; (iii) grains (staple starchy cereal foods); (iv) meat/meat alternatives (protein foods) and (v) dairy foods [3].

While the World Health Organization provides a generalised recommendation for legumes, emphasising consumption as part of a healthy diet [4], across the globe, there is substantial variability in the classification and grouping of legumes within dietary guidelines [2]. Legumes are predominately classified as part of the protein rich food group, although some countries feature them as part of the vegetable group due to their high dietary fibre, mineral and vitamin content [2]. Conversely, the Dietary Guidelines for Americans [5] and the ADG incorporate legumes in both the vegetable food group, as a good source of dietary fibre, vitamins, minerals and phytochemicals [6], and the meat alternatives group, with a protein rich nutrient profile similar to poultry, fish, lean meat and eggs [7].

While many countries include key messages for legume consumption in their dietary guidelines, there is wide incongruity between countries. In particular, some avoid the use of specific quantitative recommendations, opting instead for action words that suggest proportions, frequency and variety [1,2]. As is the case in Australia, legume consumption is promoted via the statements “consume plenty of vegetables, including different types and colour, and legumes/beans” and “consume lean meat and poultry, fish, eggs, tofu, nuts and seeds, and legumes/beans” [3]. Where countries do include quantitative recommendations, there is broad disparity in terms of units of measure, including serve sizes, portions or weight in grams [1]. Likewise, within these measures, the quantity recommended varies between countries [1,2].

Despite the inclusion of legumes in the ADG since 1992, only 28% of Australians consume legumes regularly [8], suggesting that in order to be effective, guideline recommendations need to be applied in the context of additional external measures that address previously identified barriers to intake, such as poor familiarity, lack of preparation skills and gastrointestinal discomfort [9,10]. Furthermore, studies suggest that there may be confusion around the categorisation and quantification of legume recommendations contained in the current ADG that may further hinder uptake [9].

Similar issues have been identified for whole grains and whole grain foods [11]. A recent global review of whole grain recommendations in dietary guidelines found that 44% of countries promote the consumption of whole grains, discerning these food types from more refined grains [11]. Some countries suggest half of all grain foods should be whole grain, as in the US, while others use more passive language such as “mostly” or “preferably” to promote intake [11]. In Australia, whole grain consumption is promoted via the statement consume “grain (cereal) foods, mostly wholegrain and/or high cereal fibre varieties….” [12]. Similar to legumes, whole grain consumption, globally, falls below recommendations [13], and it has been suggested that more targeted and actionable recommendations outlined in dietary guidelines may be one strategy towards improving intake [14,15]. In Denmark, whole grains are actively promoted, with consumers directed to simply “choose whole grain”, which may have contributed to increased rates of whole grain consumption over time [16].

A recent Australian consumer study that aimed to explore consumer understanding of whole grains identified a need for more definitive recommendations for whole grain food intake within the dietary guidelines [17], thus requiring a deeper understanding of the consumers’ perspective of messaging, which may encourage intake. The National Health and Medical Research Council’s coordinated review of the ADG from 2021, provides opportune timing to undertake meaningful and novel research that may optimise the wording that frames the ADG promotion of legume and whole grain intake.

This exploratory study aimed to understand Australian consumer perspectives of wording utilised in dietary guidelines, specifically focused on legumes and whole grains, in order to evaluate potential suggested wording for dietary guidelines. It was hypothesised that consumers would identify potential for improvements to the current dietary guidelines’ statements pertaining to legume and whole grain foods to enhance specificity.

## 2. Materials and Methods

A cross-sectional self-administered anonymous survey of Australian adults (aged 18 years old) was conducted using an anonymous online computer-based survey delivered via Qualtrics (Provo, UT, USA) (*n* = 314) [18]. The survey was approved by the University of Wollongong Research Ethics Committee (Ethics number: 2021/154). Individuals with a formal nutrition education or profession were excluded from participation due to the potential for bias related to enhanced levels of nutritional knowledge and higher rates of dietary guideline adherence [19,20].

The convenience and purposive recruitment of respondents was conducted via an e-survey link, promoted on social media platforms and the Grains and Legumes Nutrition Council (GLNC) eNewsletter for a period of 12 weeks from June to August 2021. Respondents were required to have internet and computer or smart phone access to complete the questionnaire. The survey was accessed via an advertised link that redirected respondents to an introductory statement and consent form, with tacit consent obtained through agreement to commence the survey. The survey included an age declaration prior to commencement and individuals with nutritional training or qualifications were excluded from participation as previously mentioned. Participation was incentivised with a random prize draw to win one of three recipe book sets valued at AUD75. During the data collection phase, additional advertisements via the promotional tools embedded in the particular social media platforms were conducted to target specific age groups and genders, aiming to increase participation across demographics.

Survey questions were developed and pilot-tested in consultation with key stakeholders, including consumers, to test construct and content validity, comprehensibility of survey questions and to establish an estimated completion time of 15–20 min. Survey questions were designed to gauge consumer understanding of the current ADG recommendations and preferences for the language used to encourage legume and whole grain intake. The final survey (Supplementary Materials S1) consisted of 20 questions and utilized an open and closed questionnaire design including a combination of multiple-choice questions, drag and drop ranking, and free text boxes for open responses. Closed multiple-choice questions were used to collect demographic characteristics such as age, gender and habitual dietary pattern (“unrestricted omnivore”, “flexitarian”, “vegetarian”, “pescatarian”, “vegan” and “other”), as well as current consumption, barriers and facilitators to legume and whole grain intake. Closed multiple choice questions were also used to determine consumer understanding of current dietary guideline messages. Respondents were provided with the current statements outlined in Guideline 2 of the ADG related to legumes and whole grains and were asked to select the statement that best explained the recommendations (Supplementary Material S1).

To explore potential new dietary guideline statements, respondents were asked to rank six statements that would encourage legume intake and five statements for whole grain food intake, where one question for each food type referred to the respondents most preferred statement and least preferred statement. The suggested messaging statements were formulated reflecting the diversity and incongruity of legume and whole grain recommendations across the globe [1,21]. The hypothetical guideline statement options included a combination of quantitative (e.g., Consume one serve of legumes daily) and qualitative recommendations (e.g., Consume mostly whole grain foods) to determine consumer preferences for the language used. An option to maintain current wording was included for comparison.

### Statistical Analysis

All survey data was exported from Qualtrics (Provo, UT, USA) to Microsoft Excel™ (Version 2202, Washington, DC, USA) for data collation. Descriptive statistics were used to provide frequency counts and percentages for demographic data, multiple choice, drag and drop ranking and Likert scale related questions. Descriptive statistics were most appropriate for this study due to the explorative nature of the analysis, and we conducted a repeated measures ANOVA with a Bonferroni correction for multiple tests in R (Version 3.6.2; The R Foundation for Statistical Computing, Vienna, Austria) for the preference-based statements relating to legumes and whole grains based only on the complete cases (275 participants). Alpha was set at 0.05. In the case of multiple-choice questions related to general understanding of the ADG, more than one response was permitted and as a result, the values presented are the proportions of respondents selecting each option. Content analysis and summary of free text responses were also undertaken.

## 3. Results

### 3.1. Respondent Demographics

The survey was attempted by 314 eligible respondents and completed in full by 275, providing a completion rate of 88%. Due to the independent nature of each question, all survey responses, inclusive of those partially completed, were included in the final analysis with varied participation numbers reflected in the results.

The majority of respondents were female (84%, *n* = 265) and aged 45 years and over (70%) (Table 1). More than half of respondents reported to follow an omnivorous dietary pattern (56%), and a quarter a flexitarian diet (23%) (Table 1).

### 3.2. Reported Consumption of Legumes and Whole Grains

Respondents most commonly reported to consume legumes several times a week (50%), daily (22%) or weekly (18%) and fewer reported consuming legumes less than once a week (Table 2). Legumes were primarily consumed as a source of protein, as an alternative to meat (82%; *n* = 224), as opposed to a serving of vegetables (44%; *n* = 121). In contrast, forty percent reported consuming legumes as both a source of protein and as a serve of vegetables (*n* = 109). Overall, just 16% of respondents identified legumes as a feature of their traditional diet (*n* = 45).

Daily whole grain consumption was common among respondents (88%; *n* = 260), most of whom reported consuming 1–2 servings (41%; *n* = 121) or 3–4 serves (42%; *n* = 123) per day, with one serving defined according to the ADG. Most commonly, respondents selected at least two reasons for the consumption of whole grains (71%; *n* = 202); predominantly as a source of dietary fibre (80%; *n* = 229) and over half enjoyed the taste of whole grains (57%; *n* = 163). Additionally, respondents reported to consume whole grains as a source of carbohydrate (45%; *n* = 129); plant-based protein (38%; *n* = 110) and as part of their traditional diet (23%; *n* = 67).

### 3.3. Consumer Understanding of the Australian Dietary Guidelines

Overall, the majority correctly identified that the ADG were developed to promote health and wellbeing (84%; *n* = 257), to serve as a healthy eating guide (68% *n* = 208) and to support healthy dietary choices (54%; *n* = 167), while half of respondents associated the ADG with reducing risk of specific chronic diseases (59%; *n* = 182) and diet-related health conditions (59%; *n* = 180). A small proportion considered the ADG to promote industry or were unsure of their purpose (12%; *n* = 38).

Commonly, the dietary guidelines were deemed applicable to healthy Australians or individuals seeking to maintain a healthy lifestyle (91%; *n* = 279); individuals seeking to lose weight (29%; *n* = 90), those with chronic disease (32%; *n* = 99) and healthcare professionals (33%; *n* = 101).

Three quarters of respondents indicated that the ADG should be followed on most days (76%; *n* = 233) as opposed to opting for strict and consistent observation (11%; *n* = 34); some or vague consideration (12%; *n* = 37) or not at all (1%; *n* = 3).

### 3.4. Australian Dietary Guidelines and Legume Recommendations

#### 3.4.1. Understanding of Legume Messaging

Almost all respondents demonstrated good understanding for the highlighted statement in Guideline 2 of the ADG pertaining to legume recommendations. As shown in Table 3, respondents most frequently selected the interpretation that “legumes can be consumed as a vegetable as well as a protein replacement for meat and eggs” (72%; *n* = 202), followed by “choose a variety of vegetable and protein foods” (61%; *n* = 171), and “legumes are an important food as they feature in two of five food groups” (47%; *n* = 132) (Table 3).

In response to an open ended question related to the two different serving sizes for legumes (75 g (½ cup) as a vegetable serving and 150 g (1 cup) as an alternative to meat), the majority reported that they found it easy to interpret (70%; *n* = 196), citing that the categorisation accompanied with a specific serving size was “clearly stated”, “easy to understand and remember” and provided a “clear size guide”. However, one fifth of respondents found the serving size and categorisation of legumes confusing (21%; *n* = 58). Some respondents stated a preference for the serving size provided as a cup measure, stating that “Cups is easier to visualise for me”, “Yes—easy to interpret if using cup measurements rather than grams”, “yes, the cup measurement means more than the weight though so put it first maybe?”. Others expressed that the differentiation between food groups and the need to measure serves is impractical and not commonly practiced. Further responses are provided in Supplementary Material S2 Table S1.

#### 3.4.2. Consumer Preferences for Legume Based Recommendations

**Figure 1:**
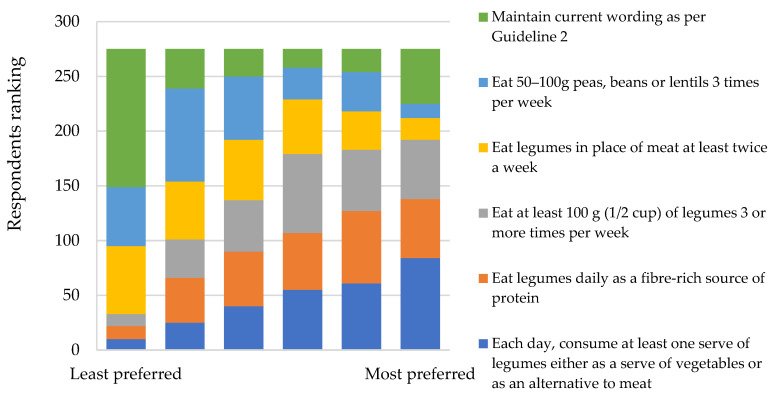
Ranking of preferred new guideline statements for the promotion of legume intake (*n* = 275).

In considering alternative wording for legume promotion in the ADG, respondents were required to rank provided statements in sequential order, whereby the most preferred statement ranked first and the least ranked sixth. Statements that emphasised daily consumption or provided a quantitative measure for intake ranked well overall (Figure 1). Overall, preference for the six statements differed significantly (*p* < 0.05). The statement “Each day, consume at least one serve of legumes either as a serve of vegetables or as an alternative to meat” was the most preferred statement, as it was selected significantly more times than all other statements, except “Eat at least 100 g (½ cup) of legumes 3 or more times per week”, where there was no significant difference. The statement “Eat 50–100 g peas, beans or lentils 3 times per week” was selected the fewest times as the most preferred statement.

When asked what would be helpful in achieving an increase in legume intake, the majority preferred legumes to feature either in their own food group or as part of the protein group (Table 4). Respondents reported a preference for recommendations that included frequency of intake and quantifiable cup measures, rather than grams (in other words, how many cups and how often). Maintaining the current guidelines was displaced by these preferred options (Table 4).

### 3.5. Australian Dietary Guidelines and Whole Grain Recommendations

#### 3.5.1. Understanding of Whole Grain Messaging

Similar to legumes, almost all respondents demonstrated good understanding of the statement in Guideline 2 related to whole grain consumption (97%, *n* = 291). The most commonly selected interpretation was “eat most (more than half) of your grain foods from whole grain choices” (53%; *n* = 157); followed by “eat from a variety of grain foods including refined and whole grains” (33%; *n* = 98) and “limit refined and low fibre grain foods” (28%; *n* = 84) (Table 5).

#### 3.5.2. Consumer Preferences for Whole Grain Recommendations

**Figure 2:**
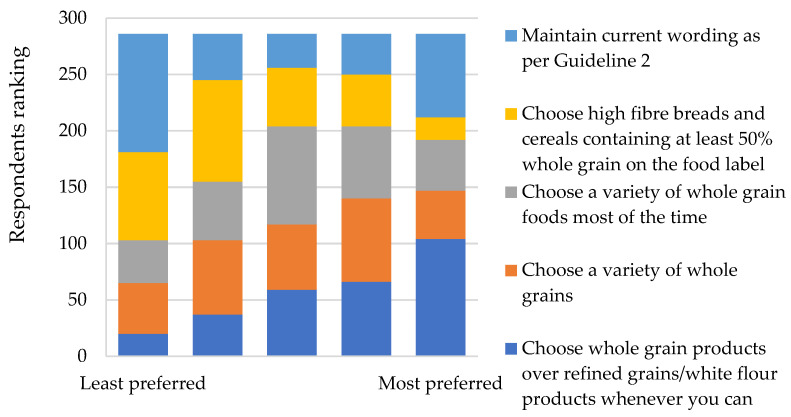
Ranking of preferred new guideline statements for the promotion of whole grain foods (*n* = 286).

To evaluate preferences towards potential new guideline statements for the promotion of whole grain foods, respondents were required to rank provided statements in sequential order, where the most preferred statement ranked first and the least ranked fifth. Similarly to legumes, there was a significant difference between all statements for whole grains (*p* < 0.05). The statement “Choose whole grain products over refined grains/white flour products whenever you can” was most preferred and had a significantly higher mean score than all other statements (*p* < 0.05) (Figure 2). Comparatively, “choose high fibre breads and cereals containing at least fifty percent whole grain in the food label” was ranked the most preferred the fewest times. Statements that emphasised the variety of whole grains also ranked well (Figure 2). Maintaining the current guideline wording received the most votes as the least preferred statement (Figure 2).

## 4. Discussion

While previous Australian studies have explored consumer perceptions and attitudes towards legumes and whole grains [10,22,23], to our knowledge, this is the first Australian study to investigate consumer understanding of, and preferences for, the language used in the ADG for the consumption of legumes and whole grains. This exploratory study provides prerequisite insight into the consumers’ perspective of the current representation of legumes and whole grains within the ADG, including preferences for categorisation, frequency and quantity of intake.

The prevalence for dietary patterns such as vegan, vegetarian or pescatarian diets reported in the present study is similar to the findings of Figueira et al. (2019); however, the notable difference here was the purposeful distinction between an unrestrictive omnivorous diet [9] and a flexitarian diet, synonymous with a more plant-based dietary pattern that allows for the flexibility to include some animal sourced foods [24]. Congruent with Figueira et al. (2019), most respondents in the current study reported to follow no specific diet, suggestive of an unrestrictive omnivorous diet. However, one quarter of respondents reported to follow a flexitarian diet, reflective of the growth seen in this dietary pattern [25,26]. The accelerating shift away from animal-sourced foods, namely dairy and red meat, as consumers endeavour to align their diet with environmental initiatives [26,27], positions legumes and whole grains as a valuable source of plant protein, dietary fibre and other key nutrients [21,28]. Recently, there has been a call on national governments for the revision of current dietary guidelines to reflect environmental sustainability objectives in a bid to integrate both environmental and population health [29,30]. If carried out correctly, a global shift towards plant-based foods is considered a dietary strategy that may prove important and beneficial for the health of humans and the planet [30,31]. Reflective of this, the Canadian dietary guidelines have recently shifted towards a plant-based diet, emphasising whole grains, legumes, nuts, seeds and fortified plant-based milk alternatives [32].

The literature shows that adherence to the ADG corresponds with higher diet quality [20] and is consequently beneficial for mood, associated with reduced risk for depression and cardiometabolic diseases [33]. Positively, our findings indicate that respondents had a good awareness and understanding of the ADG, widely acknowledging that the guidelines are aimed at healthy Australians or those who want to maintain a healthy lifestyle. Respondents expressed that the guidelines should be followed on most days; however, responses diverged regarding how strictly they should be observed. Although the results were encouraging, particularly as awareness and knowledge are considered prerequisites of behaviour change [34,35], as supported by Bandura’s social cognitive theory [36], the relationship between knowledge and actioned behaviour is complex and multifactorial and rarely equates to actual dietary change [37,38].

In this study, Guideline 2 was well interpreted for both legumes and whole grains. The current recommendations for legumes were well received, with a majority of respondents able to identify legumes as a source of vegetables and a plant-protein alternative to meat. Respondents had a good, overall understanding of the two different categorisations and serve sizes for legumes as a meat alternative or vegetable. These findings were encouraging as they explore both the comprehension and interpretation of this guideline, an important distinction given that previous studies have demonstrated that dietary guidelines may be frequently misinterpreted and poorly implemented by adults who have concurrently reported to have understood the messaging [39,40].

In the current study, the most common interpretation of Guideline 2 for grain foods was to consume most (more than half) grain foods from whole grains. Research conducted with certain population groups in the US found that despite an understanding of the whole grain statement “Make half your grains whole grains”, there were barriers to usage, related to taste, cost and identification of whole grain products [39], further demonstrating that an awareness or understanding of guidelines has been shown to rarely translate into usage [41]. These findings are not uncommon [17] and, therefore, understanding these influencers is necessary if promotion is to be meaningful [9,38].

As stated, legumes and whole grains are not widely consumed in Australia [8], yet the respondents of this study had a high reported intake, well above that of the average intake in Australia, indicated by the number of servings consumed per day rather than more precise estimations. In the case for legumes, respondents reported regular consumption, daily or at least several times a week, despite not considering them part of their traditional diet, indicating that respondents likely had a positive attitude towards legumes and possibly a good baseline knowledge of how to incorporate them into their diet [9]. In a similar manner, respondents had higher than average whole grain intakes, with 88% reporting daily consumption. From representative data of adults reporting consumption of whole grains from the National Nutrition and Physical Activity Survey (NNPAS), the median whole grain intake was 38.4 g/d, with the 48 g Daily Target Intake (DTI) reached by only 39.7% [42] of adults, with 29.1% not consuming any whole grains on the day of the survey [42]. In the current study, over half of respondents reported to enjoy the taste of legumes and whole grains, possibly contributing to high reported intakes. Taste is a strong determinant for consumption, secondary to health benefits, and is often a cited barrier to regular legume intake [9]. Consistent with Foster et al. (2020), the current study found that respondents who reported consuming whole grains, favoured these foods based on their micronutrient content, as a source of dietary fibre (80%), rather than as source of macronutrients, such as carbohydrates (45%) or protein (23%) [17]. This may have also been a function of the particular participant sample, who appear well informed regarding nutrition.

To our knowledge, there is limited published research investigating the effectiveness of messaging, provided within the ADG or any food-based guidelines, with consistency over time [41]. The findings of this exploratory study suggested a consumer preference for legume recommendations that provided an indication of a specific frequency and quantification of intake, with the statement “Each day, consume at least one serve of legumes either as a serve of vegetables or as an alternative to meat” the most preferred among respondents. The option to maintain status quo for the wording outlined in Guideline 2 was poorly received, yet, interestingly, the highest ranked statement, as previously mentioned, was quite similar to the original guideline. This popular option maintained the flexibility of legumes as a serving of vegetables or meat alternative but replaced the descriptive term “plenty of” with specified intake set at one serving daily, which was based on optimal intake modelling [43]. This preference was further supported by the popularity of other statements where quantification and frequency for intake was included in the statement. These findings are supportive of Geiger (2001), demonstrating consumer preference for specific terms relating to intake quantity and frequency in comparison to permissive terms such as “enjoy”, “balance” or “over a few days” and, likewise, with the current study, the format of the existing dietary guidelines was rated poorly [44].

The current ADG provide recommendations for serving sizes in a weighted gram measure with a corresponding cup value, whereby 75 g equates to a ½ cup [3], noting that serving size is a standardized amount of food, whereas portion size is the amount of food consumed [45]. It has been shown that consumers are better able to relate a serving size to a common cup measure rather than a weighted value, which requires higher literacy levels and scales [46]. Congruently here, respondents strongly emphasised their preference for quantifiable recommendations expressed in cup measures, stating that grams were less relevant and poorly visualised. Given the variation in legume and whole grain recommendations across regions, a standardisation of the amount promoted by health authorities and agriculture may be helpful. In the case for legumes, a recent review rationalised that 100 g cooked legumes should equate to ½ cup measure, equivalent to one serving, and further proposed this as the “standardised minimum threshold” for consumption in a single day [1,21]. Notably, in Australia, Canada and Europe, 100 g of cooked legumes satisfies the Food Standards Australia New Zealand (FSANZ) criteria required to qualify nutrient content claims for key nutrients found in legumes [21,47]. Indeed, the standardisation of this minimum threshold for consumption is appropriate for the prevention and management of chronic disease [48,49]. Determining frequency of intake for legumes over a week presents more of a challenge than serving size, as this needs to align with the cultural acceptability of a food, routine dietary patterns, and the infrastructure of the food systems [1,21]. In consideration of these factors, and the wide variety of foods available in Australia, even occasional consumption of this minimum ½ cup recommendation may enhance nutrient intake and contribute towards a healthy diet [31].

In the current study, there was less consensus regarding the categorisation of legumes. Most popular was the suggestion to feature legumes in their own food group, but this was only the case when the statement included a direct reference to quantity and frequency factors, noting that the suggestion was not popular in the absence of these indicators, and, likewise, with the suggestion to feature legumes solely in the meat alternative group. Once again, without the provision of quantity and frequency factors, the statement was poorly received; in fact, consumers expressed preference for the original wording rather than a simple reassignment of food groups in the absence of these qualifiers. Abdullah et al. (2017) suggest that classifying legumes as an “alternative” to meat may have a negative connotation, impacting consumer acceptance, and that a bolder approach is required to promote legumes as an independent food group, similar to whole grains and cereals [50].

Similarly, specific consumption guidelines for grain foods are not included in dietary guidelines, but the suggestion that most of the six servings of grain foods recommended for the 19–50 years age-group should be whole grain [12,45]. This amount would meet the 48 g DTI recommended by GLNC [51], the target developed in consultation with an expert round table and aligned with the target suggested in American Dietary Guidelines [52,53]. This recommendation is supported by evidence from systematic reviews and dose–response meta-analyses of prospective cohort studies by Aune et al. [54,55], and is supported by more recent work by Reynolds et al. [56], where each 15 g consumed assisted with risk reduction. Despite the lack of specificity, the documentation supporting the guidelines note that 70% of grains consumed in Australia are refined grains, such that a 160% increase in current whole grain consumption and a 30% decrease in refined grain (cereal) food consumption has been recommended [57]. Making this change is well supported by the food supply, with an analysis indicating one-third of breads on supermarket shelves were whole grain/wholemeal, with a median whole grain content of 20.2 g per serving (2 slices), almost half of the 48 g DTI [58,59]. This change is also supported by the outcomes of the current study, whereby respondents most preferred the dietary guideline statement “Choose whole grain products over refined grains/white flour products whenever you can”. A quantified prescription for whole grain is more difficult, due to the variability in whole grain content within foods. Instead, this must be supported by education and food labelling initiatives to simplify purchasing decisions.

### Limitations

The current study includes several limitations. Online surveys rely on convenience sampling and therefore are prone to selection bias, as respondents are required to be literate, have internet access, volunteer for participation and are more likely to have an inherent interest in nutrition [60]. Research shows that women are more likely to respond to online surveys than their male counterparts [61] and women are known to have healthier eating habits than men [21,62]. This is further exacerbated by gender-based stereotyping, whereby vegetables are considered a “female suited food”, in comparison to the masculinity associated with meat consumption [63]. Similarly, data from the NNPAS suggests that after adjusting for energy intake, adult females appeared to consume more whole grains than males, whereas slightly more males, than females, consumed no whole grain on the day of the survey (30.9% of males, 27.5% of females) [42]. The current findings, however, relate to a small female dominant sample of respondents, reporting a baseline legume and whole grain intake well above national average consumption levels and, therefore, are not representative of the Australian population [8,60].

In the instance where respondents were asked to rank the most preferable statement for potential new guidelines, it is possible that using the term “maintain current wording”, may have caused respondents to reject this option due to an association with injunctive norms [64]. Studies suggest that consumers are more likely to reject injunctive norms in preference for options that facilitate autonomy and free choice [64,65]. Furthermore, in the present study, sustainability and environmental factors were not considered part of the messaging. This was possibly a missed opportunity to explore other concepts that may enhance the messaging, given that environmental benefits are perceived important beneficial factors for the consumption of plant foods such as legumes and whole grains [1,66]. Finally, the study may have been strengthened by the contribution of communication experts to effectively interpret evidence into messaging appropriate for a range of literacy levels and thereby optimise nutrition communication strategies [67]. This is also an appropriate recommendation for the revision of dietary guidelines, where new statements may benefit from broader community consultation to gauge understanding.

There is a need for quality studies conducted to specifically evaluate the effectiveness of the dietary guidelines and their measurable contribution to population health through the translation of recommendations to dietary behaviour and change [39,40]. Finally, it is important to emphasise that the current study is an insight into components of dietary guidelines that influence consumers and does not address the challenges for aligning Australian dietary patterns with recommendations for regular legume and whole grain consumption.

## 5. Conclusions

In order to improve dietary intake for legumes and whole grains, this exploratory study highlights consumer views of suitable wording and commentary regarding the current dietary guidelines, which may help inform the review of dietary guideline statements. Findings suggest that consumers may favour legume recommendations that include quantities provided in cup measures and frequency related to daily or weekly intake. The revision of dietary recommendations may consider aligning with a standardised minimum threshold for consumption; for example, a minimum of ½ cup (100 g) cooked legumes in a single day, while offering the flexibility to consume legumes as part of the vegetable or meat alternative group. In regard to whole grains, it needs to be acknowledged that the current statement pertaining to grain (cereal) foods is reportedly ineffective and convoluted and does not direct consumers to better choices within the food group. Yet, the significance of whole grain foods within dietary patterns, evaluated by the global burden of disease research and the simplicity of emphasizing the swap to whole grain in preference to refined choices, provides the impetus to simplify and strengthen wording, and provide clarity to inform consumers to choose whole grain and high fibre food choices as a priority. Both legumes and whole grains provide economic and sustainable food choices for inclusion in dietary patterns and the range of offerings within the food supply currently would support and facilitate consumption at adequate levels to improve the health of Australians.

## Figures and Tables

**Table 1:** Demographic characteristics and dietary pattern of respondents (*n* = 314).

Demographic Variable	Count (%)
Gender	
Female	265 (84)
Male	49 (16)
Age in years	
18–24	17 (5)
25–34	31 (10)
35–44	46 (15)
45–54	76 (24)
55–64	84 (27)
65+	60 (19)
Habitual dietary pattern	
Unrestricted omnivore	176 (56)
Flexitarian	73 (23)
Vegetarian	22 (7)
Vegan	21 (7)
Pescatarian	20 (6)
Other	2 (0.6)

**Table 2:** Reported legume and whole grain intake.

	Count (%)
Reported legume consumption	*n* = 280
Several times a week	139 (50)
At least once per day	62 (22)
Approximately once a week	51 (18)
2–3 times per month	16 (6)
Irregularly (less than twice per month)	10 (4)
Never	2 (0.7)
Reported whole grain consumption	*n* = 295
3–4 serves per day	123 (42)
1–2 serves per day	121 (41)
Less than a serve per day	31 (11)
5 or more serves per day	16 (5)
I do not eat whole grain foods	3 (1)
I do not know	1 (0.3)

**Table 3:** Respondents’ interpretation of the focus of Guideline 2 for legume intake *(n* = 280) *.

Interpretation of Guideline 2 ^1^	Count (%)
Legumes can be consumed as a vegetable as well as a protein replacement for meat and eggs	202 (72)
Choose a variety of vegetable and protein foods	171 (61)
Legumes are an important food as they feature in two of the five food groups	132 (47)
Legume intake is optional	10 (4)
Eat legumes twice a day	7 (3)
Other	4 (1)
I do not know	2 (0.7)

^1^ Guideline 2 for legume intake: Enjoy a wide variety of nutritious foods from these five food groups every day including plenty of vegetables of different types and colours, and legumes/beans and lean meats and poultry, fish, eggs, tofu, nuts and seeds, and legumes/beans. * Question allowed participants to select more than one answer, consequently values presented are the proportion of respondents selecting each point and exceed 100%.

**Table 4:** Consumer preferences for guideline recommendations (*n* = 275) *.

	Count (%)
Question: If the aim is to increase legume intake, which of the following would you find most helpful in achieving this?	
Legumes feature in their own food group with recommendations for how much and how often to consume	123 (45)
Legumes feature in the meat/meat alternatives group as a source of protein, with recommendations for how much and how often to consume	61 (22)
Maintain current guideline. Recommendations are to consume legumes as part of the vegetable group and/or meat/meat alternative group	55 (20)
Legumes feature in their own food group	24 (9)
Legumes feature in the meat/meat alternatives group	12 (4)
Question: In relation to the dietary guidelines, how would you prefer the recommendations for intake to be presented?	
As a cup measure for each food/food group (e.g., consume ½ cup cooked brown rice)	136 (50)
As a suggested frequency (e.g., consume 2–3 times per week)	69 (25)
Maintain current format: As the number of serves per day for each food/ food group (e.g., consume 5 serves per day)	53 (19)
As the number of grams for each food/food group (e.g., consume 48 g whole grain)	17 (6)

* Question allowed participants to select more than one answer, consequently values presented are the proportion of respondents selecting each option.

**Table 5:** Respondents’ interpretation of the focus of Guideline 2 for whole grain intake *(n* = 295) *.

Interpretation of Guideline 2 ^1^	Count (%)
Eat most (more than half) of your grain foods from whole grain choices	157 (53)
Eat from a variety of grain foods including refined and whole grains	98 (33)
Limit refined and low fibre grain foods	84 (28)
Enjoy any kind of grain-based food	41 (14)
Eat only whole grain and/or high cereal fibre foods	41 (14)
I do not know	4 (1)

^1^ Guideline 2 for whole grain intake: Enjoy a wide variety of nutritious foods from these five food groups every day including grain (cereal) foods, mostly wholegrain and/or high cereal fibre varieties, such as breads, cereals, rice, pasta, noodles, polenta, couscous, oats, quinoa and barley. * Question allowed participants to select more than one answer, consequently values presented are the proportion of respondents selecting each option and combined exceed 100%.

## Data Availability

All data for this study is contained within the article and Supplementary Materials.

## Supplementary Materials

The following supporting information can be downloaded at: https://www.mdpi.com/article/10.3390/nu14091753/s1, Supplementary material S1: Survey Questions; Supplementary material S2: Table S1. Exemplar Quotes.
