# Supplementary material for: Consumer Understanding of the Australian Dietary Guidelines: Recommendations for Legumes and Whole Grains
Source: Nutrients. 2022 Apr 22;14(9):1753. doi: 10.3390/nu14091753 (PMC9099598; doi:10.3390/nu14091753)
Supplement: Supplementary file 1 [file nutrients-14-01753-s001.zip › Supplementary Materials S1 Survey Questions.pdf]

Supplementary Materials

Survey Questions

1. What is your gender?
   - o Female
   - o Male
   - o Other
   - o Prefer not to say

2.What is your age group?
   - o 18–24 years
   - o 25–34 years
   - o 35–44 years
   - o 45–54 years
   - o 55–64 years
   - o 65 years and older

3.Which of the following best describes your overall diet?
   - o I consume both meat and plant foods (unrestricted omnivore)
   - o I consume a, mostly, vegetarian diet but occasionally I eat meat or fish (flexitarian)
   - o I do not eat meat or fish but sometimes, I consume other animal products e.g., dairy, gelatine, honey (vegetarian)
   - o I do not eat meat but I do eat fish (pescatarian)
   - o I do not consume any animal or animal-derived products (vegan)
   - o Other

The following questions are about the <u>Australian Dietary Guidelines</u>

<u>Guideline 1</u> To achieve and maintain a healthy weight, be physically active and choose amounts of nutritious food and drinks to meet your energy needs.
<u>Guideline 2</u> Enjoy a wide variety of nutritious foods from five food groups every day
<u>Guideline 3</u> Limit intake of foods containing saturated fat, added salt, added sugars and alcohol.
<u>Guideline 4</u> Encourage, support and promote breastfeeding**.**
<u>Guideline 5</u> Care for your food; prepare and store it safely

4.Why do you think the Australian Dietary Guidelines were developed? (select all that apply)
   - o To promote the health and wellbeing of Australians
   - o To promote the food and agricultural industries
   - o To reduce the risk of diet-related health conditions such as high cholesterol and obesity
   - o To reduce the risk of chronic diseases such as heart disease and diabetes
   - o To serve as a healthy eating guide for all Australians
   - o To support healthy dietary choices
   - o I don't know

5.Who do you think the Guidelines were developed for? (Select all that apply)
   - o Individuals with chronic disease
   - o Individuals who want to lose weight
   - o Individuals who want to maintain a healthy lifestyle
   - o Health professionals
   - o All healthy Australians
   - o I don't know
   - o Other (please specify)

6.How do you think the Australian Guidelines should be followed?
   - o Strictly and consistently

- Mostly followed (e.g., most days)
- Taken into some consideration
- As a vague suggestion
- Only if I have a health condition
- Not at all

The following questions are about grain foods.

Whole grains contain all parts of the grain. (This includes the bran, germ and endosperm, and when processed into a food, these must remain in their natural proportions).Examples of wholegrains and wholegrain foods include brown rice and rolled oats, wholemeal bread and whole grain pasta. Refined grains have been processed to remove some or all of their original nutrient and fibre content (e.g., white flour, white pasta, white bread).

Please read the following Australian Dietary Guideline regarding grain (cereal) foods.

Guideline 2: Enjoy a wide variety of nutritious foods from these five food groups every day:

- Plenty of vegetables of different types and colours, and legumes/beans
- Fruit
- Grain (cereal) foods, mostly wholegrain and/or high cereal fibre varieties, such as breads, cereals, rice, pasta, noodles, polenta, couscous, oats, quinoa and barley
- Lean meats and poultry, fish, eggs, tofu, nuts and seeds, and legumes/beans
- Milk, yoghurt, cheese and/or their alternatives, mostly reduced fat
- And drink plenty of water

(Sourced from https://www.eatforhealth.gov.au/guidelines/australian-dietary-guidelines-1–5)

7.In your opinion, what is the focus of the highlighted statement above? (Select all that apply)

- Enjoy any kind of grain-based food
- Eat from a variety of grain foods including refined and whole grains
- Eat most (more than half) of your grain foods from whole grain choices
- Limit refined and low fibre grain foods
- Eat only whole grain and/or high cereal fibre foods
- I don't know

The following questions are about whole grain foods.

8.Which of the following do you think classifies a food as a 'whole grain food'? (Select all that apply)

- Products labelled 'wholemeal' such as wholemeal bread or wholemeal pasta
- Any product that contains seeds
- Products that contain more whole grain than refined grain ingredients
- Unprocessed grain foods such as oats, brown rice, freekeh and quinoa
- I don't know

9.How confident do you feel identifying a whole grain food/ product?

- Very confident
- Fairly confident
- Confident
- Slightly confident
- Not at all confident

10.How many serves of whole grains do you eat in a single day? (One serve equates to 1 slice (40g) of wholemeal or whole grain bread; ½ cup cooked oats; 2/3 cup whole grain cereal or ½ cup brown rice.)

- 5 or more serves per day
- 3–4 serves per day

- 1–2 serves per day
- Less than a serve per day
- I don't know
- I do not eat whole grain foods

11a. Why do you choose to eat whole grains? (Select all that apply)

- As a source of carbohydrate
- As a source of plant protein
- As a source of dietary fibre
- I enjoy the taste
- They are part of my traditional diet
- Other

11b. Why do you choose whole grains infrequently or not at all? (Select all that apply)

- I have never tried them before
- I do not like the taste
- My family members/ children don't like them
- I do not know how to cook/ prepare them
- No reason, they are just not a part of my meals
- They cause discomfort/ wind/ bloating/ other health issues
- Other

12.The following are potential new guideline statements to help consumers understand the types of grain foods they need to eat. Please rank the following 5 statements from 1 to 5, where '1' represents <u>the most</u> preferred statement and '5' is the statement that is <u>the least</u> preferred: *(Please drag the statement into ranked position)*

- Choose a variety of whole grains
- Choose a variety of whole grain foods most of the time
- Choose whole grain products over refined grains/white flour products whenever you can
- Choose high fibre breads and cereals containing at least 50% whole grain on the food label
- Maintain current wording (Guideline 2: Grain (cereal) foods, mostly wholegrain and/or high cereal fibre varieties, such as breads, cereals, rice, pasta, noodles, polenta, couscous, oats, quinoa and barley)

The following questions are about legumes.

Legumes such as lentils, chickpeas, beans, peas and lupins are the edible pod, fruit or seeds of the Fabaceae plant family. Legumes come in a variety of shapes, colours and sizes and may be consumed in a variety of forms including whole or split (dried, frozen or canned) or ground into flours. Some commonly consumed legumes include lentils, chickpeas, baked beans, kidney beans, green peas and hummus.

Guideline 2: Enjoy a wide variety of nutritious foods from these five food groups every day:

- Plenty of vegetables of different types and colours, and legumes/beans
- Fruit
- Grain (cereal) foods, mostly wholegrain and/or high cereal fibre varieties, such as breads, cereals, rice, pasta, noodles, polenta, couscous, oats, quinoa and barley
- Lean meats and poultry, fish, eggs, tofu, nuts and seeds, and legumes/beans
- Milk, yoghurt, cheese and/or their alternatives, mostly reduced fat
- And drink plenty of water

(Sourced from https://www.eatforhealth.gov.au/guidelines/australian-dietary-guidelines-1–5)

13.Legumes/ beans are also mentioned in Guideline 2. What do you think is the focus of the highlighted sections in Guideline 2 above? (Select all that apply)

- Choose a variety of vegetable and protein foods
- Eat legumes twice a day

- Legumes can be consumed as a vegetable as well as a protein replacement for meat and eggs
- Legumes are an important food as they feature in two of the five food groups
- Legume intake is optional
- I don't know
- Other (please type your answer)

What is a 'serve' of legumes?

It depends on whether you are eating legumes as a vegetable or as an alternative to meat:

- As a vegetable, one serve is 75 grams (1/2 cup) of cooked, dried or canned beans, chickpeas or lentils (no added salt)
- As a meat alternative, one serve is 150 grams (1 cup) of cooked, dried or canned beans, chickpeas or lentils (no added salt)

14.What are your thoughts on the two different serve suggestions for legumes above. (Do you find it easy to interpret?)

Please type your response here ________________________

15.How often do you consume legumes (such as chickpeas, beans, lentils and peas)?

- At least once per day
- Several times a week
- Approximately once a week
- 2–3 times per month
- Irregularly (less than twice per month)
- Never

16a. Why do you choose to eat legumes? (Select all that apply)

- As a source of protein
- As an alternative to meat
- As a serve of vegetables
- I enjoy the taste
- They are part of my traditional diet
- Other

16b. Why do you choose legumes infrequently or not at all? (Select *all that apply)*

- I have never tried them before
- I do not like the taste
- My family members/ children don't like them
- I do not know how to cook/ prepare them
- I don't have time to cook/prepare them
- No reason, they are just not a part of my meals
- They cause discomfort/ wind/ bloating/ other health issues
- Other

17.The following are potential new guideline statements to help consumers understand legumes. Please rank the following 6 statements from 1 to 6, where '1' represents the statement that is mostly likely to increase your intake of legumes and '6' is the statement that is least likely. *(Please drag the statement into ranked position)*

- Each day, consume at least one serve of legumes either as a serve of vegetables or as an alternative to meat
- Eat legumes daily as a fibre-rich source of protein
- Eat at least 100g (1/2 cup) of legumes 3 or more times per week
- Eat legumes in place of meat at least twice a week
- Eat 50–100g peas, beans or lentils 3 times per week
- Maintain current wording (Guideline 2: - Plenty of vegetables of different types and colours, and legumes/beans. - Lean meats and poultry, fish, eggs, tofu, nuts and seeds, and legumes/beans)

18.If the aim is to increase legume intake, which of the following would you find most helpful in achieving this?

- Legumes feature in their own food group

- Legumes feature in their own food group with recommendations for how much and how often to consume
- Legumes feature in the meat/ meat alternatives group
- Legumes feature in the meat/ meat alternatives group as a source of protein, with recommendations for how much and how often to consume
- Maintain current guideline. Recommendations are to consume legumes as part of the vegetable group and /or meat/ meat alternative group

19. In relation to the dietary guidelines, how would you prefer the recommendations for intake to be presented?
    - As the number of grams for each food/ food group (e.g consume 48g whole grain)
    - As a cup measure for each food/ food group (e.g consume ½ cup cooked brown rice)
    - As a suggested frequency (e.g consume 2–3 times per week)
    - Maintain current format: As the number of serves per day for each food/ food group (e.g consume 5 serves per day)
20. Would you like to be entered into the draw to win 1 of 3 Ottolenghi book sets valued at $75?
    - Yes
    - No
