# Supplementary material for: Consumer Understanding of the Australian Dietary Guidelines: Recommendations for Legumes and Whole Grains
Source: Nutrients. 2022 Apr 22;14(9):1753. doi: 10.3390/nu14091753 (PMC9099598; doi:10.3390/nu14091753)
Supplement: Supplementary file 1 [file nutrients-14-01753-s001.zip › Supplementary Materials S2 Exemplar Quotes Table.pdf]

Supplementary Materials

Table S1. Exemplar quotes from the following question: What are your thoughts on the two different serve suggestions for legumes above? Do you find it easy to interpret? (n = 280).

Count (%)		Exemplar quote
Yes (easy)	196 (70)	"Yes, it is easy to interrupt and understand using legumes as a vegetable serve and a meat alternative"<br>"Simply described - half a cup as one of your veg serves & one cup as one of your meat serves"<br>"I like having it expressed in cup sizes"<br>"It is useful how they have both a gram and volume measurement. The cup measurement is much easier to visualise, whereas saying 75g/150g really doesn't mean much.."
No (Not easy)	57 (20)	"Not really, I had to read it a few times to comprehend the meaning"<br>"A bit confusing as to what they are as a vegetable and how they differ as a protein"<br>"This is a confusing message for the general public"<br>"Needs to be clarified. Purpose of consuming Legumes needs to be clearer"<br>"Too prescriptive. Uninspiring"
Neutral responses	26 (10)	"I don't measure out my serve, I just eat as many legumes as I feel like regardless of them being a vegetable or protein replacement"<br>"I wouldn't really pay attention to the amount. They are nutritious so I would eat as much as I wish"
